# Supplementary material for: Hippocampal hub failure is linked to long-term memory impairment in anti-NMDA-receptor encephalitis: insights from structural connectome graph theoretical network analysis
Source: J Neurol. 2024 Jul 8;271(9):5886–98. doi: 10.1007/s00415-024-12545-4 (PMC11377655; doi:10.1007/s00415-024-12545-4)
Supplement: Supplementary file 1 — Supplementary file1 (DOCX 6146 KB) [file 415_2024_12545_MOESM1_ESM.docx]

Supplementary Material:

**Hippocampal hub failure is linked to long-term memory impairment in**

**anti-NMDA-receptor encephalitis -**

**Insights from structural connectome graph theoretical network analysis**

# *Nodular und Modular Graph Metrics of all nodes for all parameters*

**Supplementary Table S1.** Node Strength for every MTL and DMN region

Values are displayed as mean ± standard deviation. Use of L. and R. denotes left and right, respectively

| **Anatomical Region** | **HC** | | | **NMDA** | | | **T-value** | **p-value** |
| --- | --- | --- | --- | --- | --- | --- | --- | --- |
| **L.EC** | 1.67 · 10^5^ | ± | 0.42 · 10^5^ | 1.59 · 10^5^ | ± | 0.36 · 10^5^ | 1.070 | 0.281 |
| **L.HI** | 3.64 · 10^5^ | ± | 0.51 · 10^5^ | 3.45 · 10^5^ | ± | 0.51 · 10^5^ | 2.021 | 0.049* |
| **L.PHIG** | 1.73 · 10^5^ | ± | 0.27 · 10^5^ | 1.70 · 10^5^ | ± | 0.30 · 10^5^ | 0.506 | 0.609 |
| **R.EC** | 1.43 · 10^5^ | ± | 0.34 · 10^5^ | 1.46 · 10^5^ | ± | 0.35 · 10^5^ | -0.409 | 0.682 |
| **R.HI** | 3.44 · 10^5^ | ± | 0.42 · 10^5^ | 3.26 · 10^5^ | ± | 0.39 · 10^5^ | 2.479 | 0.013* |
| **R.PHIG** | 1.69 · 10^5^ | ± | 0.24 · 10^5^ | 1.63 · 10^5^ | ± | 0.26 · 10^5^ | 1.311 | 0.192 |
| **L.CACG** | 1.41 · 10^5^ | ± | 0.28 · 10^5^ | 1.42 · 10^5^ | ± | 0.27 · 10^5^ | -0.160 | 0.870 |
| **L.CMFG** | 2.56 · 10^5^ | ± | 0.36 · 10^5^ | 2.52 · 10^5^ | ± | 0.28 · 10^5^ | 0.595 | 0.540 |
| **L.IPG** | 3.57 · 10^5^ | ± | 0.49 · 10^5^ | 3.64 · 10^5^ | ± | 0.43 · 10^5^ | -0.834 | 0.410 |
| **L.MOFG** | 3.76 · 10^5^ | ± | 0.71 · 10^5^ | 3.83 · 10^5^ | ± | 0.61 · 10^5^ | -0.514 | 0.614 |
| **L.PCG** | 1.69 · 10^5^ | ± | 0.27 · 10^5^ | 1.72 · 10^5^ | ± | 0.25 · 10^5^ | -0.600 | 0.549 |
| **L.RACG** | 1.95 · 10^5^ | ± | 0.33 · 10^5^ | 2.02 · 10^5^ | ± | 0.37 · 10^5^ | -1.078 | 0.282 |
| **L.RMFG** | 4.71 · 10^5^ | ± | 0.73 · 10^5^ | 4.79 · 10^5^ | ± | 0.61 · 10^5^ | -0.618 | 0.537 |
| **L.PCU** | 4.06 · 10^5^ | ± | 0.56 · 10^5^ | 4.20 · 10^5^ | ± | 0.42 · 10^5^ | -1.635 | 0.100 |
| **R.CACG** | 1.49 · 10^5^ | ± | 0.24 · 10^5^ | 1.50 · 10^5^ | ± | 0.22 · 10^5^ | -0.412 | 0.685 |
| **R.CMFG** | 2.34 · 10^5^ | ± | 0.35 · 10^5^ | 2.36 · 10^5^ | ± | 0.22 · 10^5^ | -0.254 | 0.799 |
| **R.IPG** | 4.14 · 10^5^ | ± | 0.49 · 10^5^ | 4.13 · 10^5^ | ± | 0.55 · 10^5^ | 0.129 | 0.899 |
| **R.MOFG** | 3.04 · 10^5^ | ± | 0.48 · 10^5^ | 3.09 · 10^5^ | ± | 0.49 · 10^5^ | -0.505 | 0.609 |
| **R.PCG** | 1.68 · 10^5^ | ± | 0.26 · 10^5^ | 1.70 · 10^5^ | ± | 0.22 · 10^5^ | -0.621 | 0.541 |
| **R.RACG** | 1.50 · 10^5^ | ± | 0.26 · 10^5^ | 1.59 · 10^5^ | ± | 0.25 · 10^5^ | -1.975 | 0.056 |
| **R.RMFG** | 4.74 · 10^5^ | ± | 0.65 · 10^5^ | 4.79 · 10^5^ | ± | 0.69 · 10^5^ | -0.441 | 0.680 |
| **R.PCU** | 4.02 · 10^5^ | ± | 0.58 · 10^5^ | 4.26 · 10^5^ | ± | 0.50 · 10^5^ | -2.434 | 0.013* |

**Supplementary Table S2.** Nodal Betweenness Centrality for every MTL and DMN region

Values are displayed as mean ± standard deviation. Use of L. and R. denotes left and right, respectively

| **Anatomical Region** | **HC** | | | **NMDA** | | | **T-value** | **p-value** |
| --- | --- | --- | --- | --- | --- | --- | --- | --- |
| **L.EC** | 15.2 | ± | 20.2 | 9.5 | ± | 11.4 | 1.915 | 0.042* |
| **L.HI** | 108.7 | ± | 50.7 | 98.0 | ± | 60.9 | 1.048 | 0.289 |
| **L.PHIG** | 1.4 | ± | 6.3 | 4.5 | ± | 10.7 | -1.943 | 0.054 |
| **R.EC** | 6.7 | ± | 9.0 | 7.9 | ± | 10.9 | -0.655 | 0.512 |
| **R.HI** | 92.6 | ± | 44.1 | 80.3 | ± | 49.4 | 1.437 | 0.151 |
| **R.PHIG** | 5.2 | ± | 12.5 | 2.5 | ± | 5.5 | 1.543 | 0.126 |
| **L.CACG** | 2.6 | ± | 11.8 | 13.6 | ± | 49.7 | -1.675 | 0.108 |
| **L.CMFG** | 10.5 | ± | 14.8 | 5.3 | ± | 12.2 | 2.105 | 0.037* |
| **L.IPG** | 98.3 | ± | 59.7 | 102.4 | ± | 59.5 | -0.383 | 0.703 |
| **L.MOFG** | 130.7 | ± | 126.6 | 128.3 | ± | 127.9 | 0.103 | 0.919 |
| **L.PCG** | 22.9 | ± | 23.1 | 21.0 | ± | 24.8 | 0.422 | 0.685 |
| **L.RACG** | 5.0 | ± | 13.7 | 9.6 | ± | 20.9 | -1.417 | 0.172 |
| **L.RMFG** | 163.2 | ± | 70.2 | 161.6 | ± | 71.7 | 0.128 | 0.895 |
| **L.PCU** | 120.4 | ± | 93.8 | 120.6 | ± | 110.2 | -0.011 | 0.992 |
| **R.CACG** | 3.3 | ± | 7.6 | 13.8 | ± | 49.5 | -1.630 | 0.130 |
| **R.CMFG** | 5.9 | ± | 24.5 | 10.9 | ± | 33.4 | -0.936 | 0.337 |
| **R.IPG** | 188.1 | ± | 63.0 | 169.0 | ± | 80.9 | 1.442 | 0.155 |
| **R.MOFG** | 112.7 | ± | 129.6 | 113.5 | ± | 130.5 | -0.036 | 0.972 |
| **R.PCG** | 15.3 | ± | 22.4 | 16.0 | ± | 20.3 | -0.196 | 0.843 |
| **R.RACG** | 2.7 | ± | 12.0 | 1.1 | ± | 3.0 | 1.041 | 0.402 |
| **R.RMFG** | 144.3 | ± | 66.3 | 141.1 | ± | 68.9 | 0.263 | 0.788 |
| **R.PCU** | 119.6 | ± | 97.7 | 121.0 | ± | 113.0 | -0.077 | 0.933 |

**Supplementary Table S3.** Nodal Average Path Length for every MTL and DMN region

Values are displayed as mean ± standard deviation. Use of L. and R. denotes left and right, respectively

| **Anatomical Region** | **HC** | | | **NMDA** | | | **T-value** | **p-value** |
| --- | --- | --- | --- | --- | --- | --- | --- | --- |
| **L.EC** | 4.1 · 10^-4^ | ± | 0.6 · 10^-4^ | 4.3 · 10^-4^ | ± | 0.7 · 10^-4^ | -1.469 | 0.147 |
| **L.HI** | 3.3 · 10^-4^ | ± | 0.3 · 10^-4^ | 3.5 · 10^-4^ | ± | 0.5 · 10^-4^ | -2.391 | 0.017* |
| **L.PHIG** | 3.8 · 10^-4^ | ± | 0.4 · 10^-4^ | 3.9 · 10^-4^ | ± | 0.4 · 10^-4^ | -1.532 | 0.128 |
| **R.EC** | 4.4 · 10^-4^ | ± | 0.7 · 10^-4^ | 4.4 · 10^-4^ | ± | 1.0 · 10^-4^ | 0.106 | 0.924 |
| **R.HI** | 3.4 · 10^-4^ | ± | 0.4 · 10^-4^ | 3.6 · 10^-4^ | ± | 0.4 · 10^-4^ | -1.953 | 0.052 |
| **R.PHIG** | 3.9 · 10^-4^ | ± | 0.4 · 10^-4^ | 4.0 · 10^-4^ | ± | 0.4 · 10^-4^ | -2.314 | 0.026* |
| **L.CACG** | 3.6 · 10^-4^ | ± | 0.4 · 10^-4^ | 3.7 · 10^-4^ | ± | 0.4 · 10^-4^ | -0.728 | 0.473 |
| **L.CMFG** | 2.9 · 10^-4^ | ± | 0.3 · 10^-4^ | 3.0 · 10^-4^ | ± | 0.3 · 10^-4^ | -1.238 | 0.215 |
| **L.IPG** | 2.7 · 10^-4^ | ± | 0.3 · 10^-4^ | 2.7 · 10^-4^ | ± | 0.2 · 10^-4^ | 0.467 | 0.646 |
| **L.MOFG** | 3.0 · 10^-4^ | ± | 0.4 · 10^-4^ | 3.1 · 10^-4^ | ± | 0.4 · 10^-4^ | -0.596 | 0.547 |
| **L.PCG** | 3.7 · 10^-4^ | ± | 0.4 · 10^-4^ | 3.8 · 10^-4^ | ± | 0.4 · 10^-4^ | -0.713 | 0.466 |
| **L.RACG** | 3.6 · 10^-4^ | ± | 0.4 · 10^-4^ | 3.6 · 10^-4^ | ± | 0.4 · 10^-4^ | 0.229 | 0.822 |
| **L.RMFG** | 2.6 · 10^-4^ | ± | 0.3 · 10^-4^ | 2.6 · 10^-4^ | ± | 0.3 · 10^-4^ | -0.502 | 0.618 |
| **L.PCU** | 2.8 · 10^-4^ | ± | 0.3 · 10^-4^ | 2.8 · 10^-4^ | ± | 0.2 · 10^-4^ | 1.392 | 0.160 |
| **R.CACG** | 3.5 · 10^-4^ | ± | 0.4 · 10^-4^ | 3.6 · 10^-4^ | ± | 0.4 · 10^-4^ | -1.439 | 0.150 |
| **R.CMFG** | 3.0 · 10^-4^ | ± | 0.3 · 10^-4^ | 3.0 · 10^-4^ | ± | 0.3 · 10^-4^ | -0.328 | 0.747 |
| **R.IPG** | 2.5 · 10^-4^ | ± | 0.2 · 10^-4^ | 2.5 · 10^-4^ | ± | 0.2 · 10^-4^ | -0.387 | 0.708 |
| **R.MOFG** | 3.1 · 10^-4^ | ± | 0.3 · 10^-4^ | 3.2 · 10^-4^ | ± | 0.3 · 10^-4^ | -1.105 | 0.272 |
| **R.PCG** | 3.8 · 10^-4^ | ± | 0.4 · 10^-4^ | 3.8 · 10^-4^ | ± | 0.4 · 10^-4^ | 0.186 | 0.861 |
| **R.RACG** | 4.0 · 10^-4^ | ± | 0.4 · 10^-4^ | 3.9 · 10^-4^ | ± | 0.4 · 10^-4^ | 0.447 | 0.654 |
| **R.RMFG** | 2.6 · 10^-4^ | ± | 0.2 · 10^-4^ | 2.6 · 10^-4^ | ± | 0.2 · 10^-4^ | -0.844 | 0.396 |
| **R.PCU** | 2.8 · 10^-4^ | ± | 0.3 · 10^-4^ | 2.7 · 10^-4^ | ± | 0.2 · 10^-4^ | 1.574 | 0.125 |

**Supplementary Table S4.** Nodal CC for every MTL and DMN region

Values are displayed as mean ± standard deviation. Use of L. and R. denotes left and right, respectively

| **Anatomical Region** | **HC** | | | **NMDA** | | | **T-value** | **p-value** |
| --- | --- | --- | --- | --- | --- | --- | --- | --- |
| **L.EC** | 8.4 | ± | 2.0 | 8.6 | ± | 1.6 | -0.549 | 0.577 |
| **L.HI** | 3.1 | ± | 0.5 | 3.0 | ± | 0.5 | 0.628 | 0.534 |
| **L.PHIG** | 7.2 | ± | 1.7 | 7.5 | ± | 1.6 | -1.116 | 0.258 |
| **R.EC** | 9.3 | ± | 1.9 | 9.3 | ± | 1.9 | 0.167 | 0.875 |
| **R.HI** | 3.1 | ± | 0.5 | 3.0 | ± | 0.5 | 0.306 | 0.763 |
| **R.PHIG** | 6.8 | ± | 1.6 | 7.1 | ± | 1.6 | -1.113 | 0.261 |
| **L.CACG** | 3.6 | ± | 0.6 | 3.6 | ± | 0.6 | -0.576 | 0.561 |
| **L.CMFG** | 4.9 | ± | 1.0 | 5.1 | ± | 0.9 | -1.174 | 0.238 |
| **L.IPG** | 4.3 | ± | 0.8 | 4.3 | ± | 0.7 | 0.041 | 0.969 |
| **L.MOFG** | 5.6 | ± | 1.2 | 5.7 | ± | 1.2 | -0.677 | 0.506 |
| **L.PCG** | 3.1 | ± | 0.6 | 3.1 | ± | 0.5 | 0.053 | 0.960 |
| **L.RACG** | 3.9 | ± | 0.7 | 4.1 | ± | 0.7 | -1.666 | 0.095 |
| **L.RMFG** | 4.9 | ± | 1.1 | 5.1 | ± | 0.9 | -1.061 | 0.298 |
| **L.PCU** | 2.6 | ± | 0.5 | 2.7 | ± | 0.4 | -1.295 | 0.196 |
| **R.CACG** | 3.6 | ± | 0.7 | 3.5 | ± | 0.6 | 0.273 | 0.791 |
| **R.CMFG** | 5.1 | ± | 1.2 | 5.2 | ± | 0.9 | -0.241 | 0.815 |
| **R.IPG** | 4.4 | ± | 0.8 | 4.4 | ± | 0.8 | 0.123 | 0.910 |
| **R.MOFG** | 5.9 | ± | 1.2 | 6.1 | ± | 1.2 | -0.906 | 0.360 |
| **R.PCG** | 3.2 | ± | 0.6 | 3.1 | ± | 0.6 | 0.103 | 0.918 |
| **R.RACG** | 4.0 | ± | 0.8 | 4.1 | ± | 0.7 | -1.257 | 0.204 |
| **R.RMFG** | 4.7 | ± | 1.0 | 4.9 | ± | 0.8 | -1.302 | 0.191 |
| **R.PCU** | 2.6 | ± | 0.5 | 2.7 | ± | 0.5 | -1.044 | 0.296 |

## **Abbreviations of Anatomical Regions**

| **Abbreviation** | **Anatomical Region** |
| --- | --- |
| **EC** | entorhinal cortex |
| **HI** | hippocampus |
| **PHIG** | parahippocampal gyrus |
| **CACG** | caudal anterior cingulate |
| **CMFG** | caudal middle frontal gyrus |
| **IPG** | inferior parietal gyrus |
| **MOFG** | medial orbitofrontal gyrus |
| **PCG** | postcentral gyrus |
| **RACG** | rostral anterior cingulate |
| **RMFG** | rostral middle frontal gyrus |
| **PCU** | precuneus |
| **MTL** | medial temporal lobe |
| **DMN** | default-mode network |

**Supplementary Table S5.** Modular MTL and DMN graph metrics in NMDAR encephalitis and HC.

Mean (sd) values are given across all subjects per group; HC = healthy controls; anti-NMDAR encephalitis = anti-N-methyl-D-aspartate receptor encephalitis; BC = betweenness centrality; CC = clustering coefficient; APL = average shortest path length.

|  |  | **HC** | | **NMDAR encephalitis** | | ***p*-value** |
| --- | --- | --- | --- | --- | --- | --- |
|  |  | *mean* | *sd* | *mean* | *sd* |  |
| **Node Strength** | Left MTL | 23.5 · 10^4^ | 4.2 · 10^4^ | 22.5 · 10^4^ | 3.0 · 10^4^ | 0.09 |
|  | Right MTL | 21.9 · 10^4^ | 2.5 · 10^4^ | 21.1 · 10^4^ | 2.2 · 10^4^ | 0.09 |
|  | Left DMN | 29.6 · 10^4^ | 2.7 · 10^4^ | 30.2 · 10^4^ | 2.1 · 10^4^ | 0.23 |
|  | Right DMN | 28.7 · 10^4^ | 2.6 · 10^4^ | 29.3 · 10^4^ | 2.2 · 10^4^ | 0.17 |
| **BC** | Left MTL | 41.8 | 18.5 | 37.3 | 21.7 | 0.23 |
|  | Right MTL | 34.8 | 17.2 | 30.2 | 16.7 | 0.14 |
|  | Left DMN | 69.2 | 23.6 | 70.3 | 25.0 | 0.80 |
|  | Right DMN | 74.0 | 24.3 | 73.3 | 30.4 | 0.90 |
| **CC** | Left MTL | 6.2 · 10^-3^ | 1.2 · 10^-3^ | 6.4 · 10^-3^ | 1.1 · 10^-3^ | 0.48 |
|  | Right MTL | 6.4 · 10^-3^ | 1.2 · 10^-3^ | 6.5 · 10^-3^ | 1.1 · 10^-3^ | 0.69 |
|  | Left DMN | 4.1 · 10^-3^ | 0.7 · 10^-3^ | 4.2 · 10^-3^ | 0.6 · 10^-3^ | 0.34 |
|  | Right DMN | 4.2 · 10^-3^ | 0.7 · 10^-3^ | 4.3 · 10^-3^ | 0.6 · 10^-3^ | 0.50 |
| **APL** | Left MTL | 3.7 · 10^-4^ | 0.4 · 10^-4^ | 3.9 · 10^-4^ | 0.5 · 10^-4^ | 0.05 |
|  | Right MTL | 3.9 · 10^-4^ | 0.4 · 10^-4^ | 4.0 · 10^-4^ | 0.5 · 10^-4^ | 0.25 |
|  | Left DMN | 3.1 · 10^-4^ | 0.2 · 10^-4^ | 3.2 · 10^-4^ | 0.2 · 10^-4^ | 0.71 |
|  | Right DMN | 3.2 · 10^-4^ | 0.2 · 10^-4^ | 3.2 · 10^-4^ | 0.2 · 10^-4^ | 0.75 |

# *Global Graph Metrics*

**Table S6. Network characterization with global and modular graph metrics in NMDAR encephalitis and HC.**

Mean(sd) values are given across all subjects per group with connection densities from 10% to 55%; range values are given as mean(sd) for 10% density and 55% density; HC = healthy controls; anti-NMDAR encephalitis = anti-N-methyl-D-aspartate receptor encephalitis; sigma = small-worldness; Eglob = global efficiency; CCglob = global clustering coefficient calculated as the mean CC across all nodes.

|  |  | **HC** | **NMDAR encephalitis** | **p-value** |
| --- | --- | --- | --- | --- |
| **Global** |  |  |  |  |
| **Sigma** | Mean(sd) | 1.86 (0.74) | 1.87 (0.75) | 0.45 |
|  | Range | 3.58 (0.15) – 1.14 (0.04) | 3.60 (0.18) – 1.13 (0.05) |  |
| **Eglob** | Mean(sd) | 1.396 (69) | 1.393 (88) | 0.83 |
|  | Range | 1.4 · 10^3^ (70) – 1.4 · 10^3^ (70) | 1.4 · 10^3^ (89) – 1.4 · 10^3^ (89) |  |
| **CCglob** | Mean(sd) | 0.012 (0.006) | 0.013 (0.006) | 0.30 |
|  | Range | 0.031 (0.005) – 0.004 (0.001) | 0.032 (0.004) – 0.004 (0.001) |  |

**Figure S1**


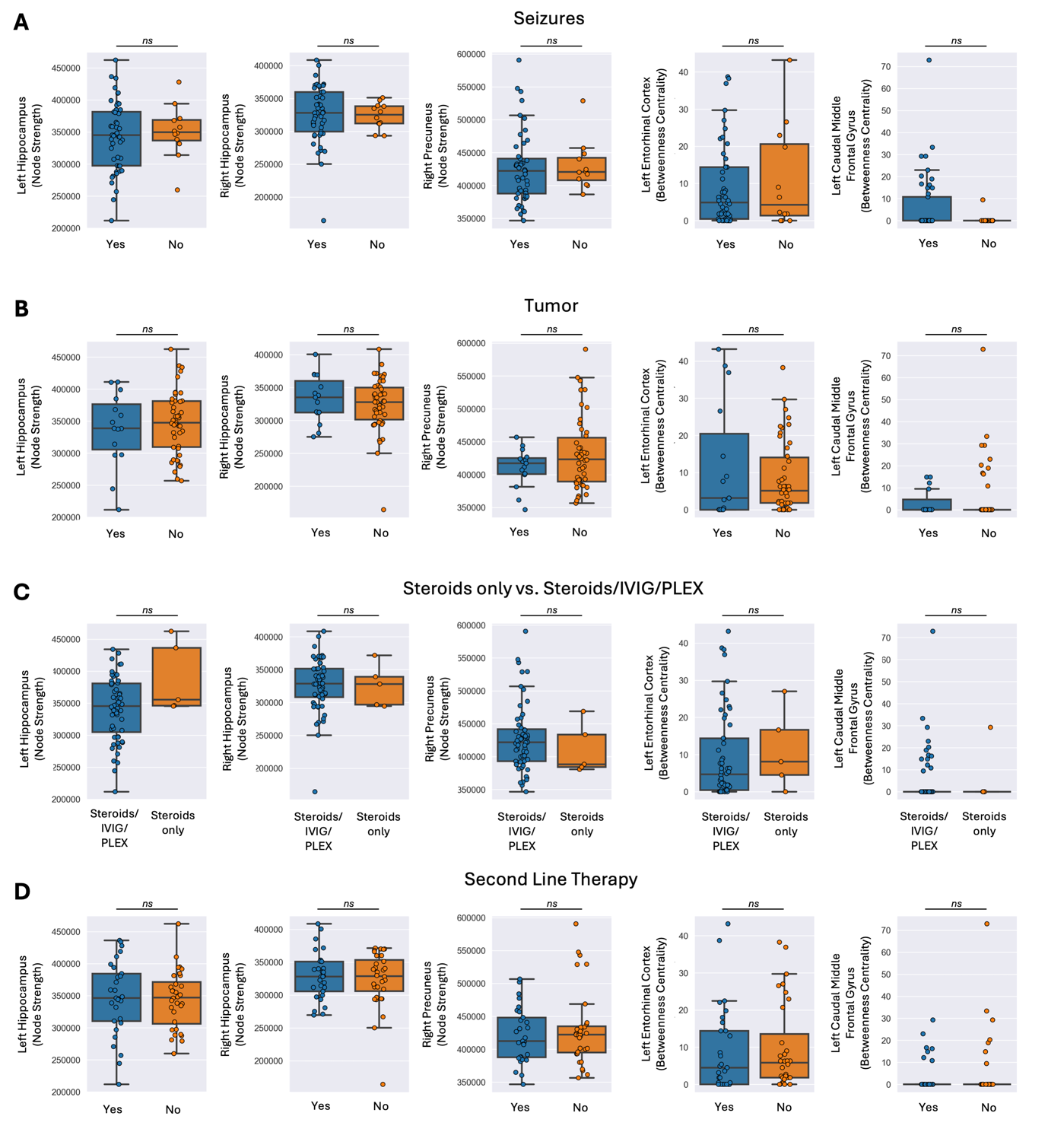


**Figure S1. Clinical subgroup analyses for graph analysis parameters**

Structural connectome-based graph analysis parameters did not show differences between subgroups based on clinical parameters regarding **A** ongoing seizures, **B** presence of a tumor, **C** initial therapy with steroids only or with combination of steroids, plasmapheresis (PLEX) or immunoadsorption and/or intravenous immunoglobulins (IVIG), and **D** administration of second-line therapy. Analyses were all exploratory in nature. Subgroups were not balanced, and subgroup assignment regarding first and second line therapy might be affected by treatment bias effects. Graph analysis parameters investigated were left hippocampal node strength, right hippocampal node strength, right precuneus node strength, left entorhinal cortex betweenness centrality, and left caudal middle frontal gyrus betweenness centrality.
